# Late peripheral facial paralysis after COVID-19: a rapid systematic review and two case reports

**DOI:** 10.1590/1678-9199-JVATITD-2022-0020

**Published:** 2022-10-17

**Authors:** Thalitta Mendes Cavalcante, Vanessa Terezinha Gubert, Carolina de Deus Lima, Larissa Anjos Luciano, Mariana Garcia Croda, James Venturini, Antonio Luiz Dal Bello Gasparoto, Wellyngton Matheus Souza Santiago, Ana Rita Coimbra Motta-Castro, Fernanda Paes Reis, Ana Paula da Costa Marques, Aline Pedroso Lorenz, Wellington Santos Fava, Marina Castilhos Souza Umaki Zardin, Cláudia Elizabeth Volpe Chaves, Gabriel Pereira Braga, Anamaria Mello Miranda Paniago, Sandra Maria do Valle Leone de Oliveira

**Affiliations:** 1 School of Medicine, Federal University of Mato Grosso do Sul (UFMS), Campo Grande, MS, Brazil.; 2 Graduate Program in Infectious and Parasitic Diseases, Federal University of Mato Grosso do Sul (UFMS), Campo Grande, MS, Brazil.; 3 Maria Aparecida Pedrossian University Hospital (UFMS/EBSERH), Campo Grande, MS, Brazil.; 4 Fiocruz Mato Grosso do Sul, Oswaldo Cruz Foundation, Campo Grande, MS, Brazil.; 5 Institute of Biosciences, Federal University of Mato Grosso do Sul (UFMS), Campo Grande, MS, Brazil.; 6 Central Laboratory of Public Health (LACEN) of Mato Grosso do Sul, Campo Grande, MS, Brazil.; 7 Rosa Pedrossian Regional Hospital (HRMS), State Secretariat of Health, Campo Grande, MS, Brazil.

**Keywords:** SARS-CoV-2 infection, Peripheral facial paralysis, Post-COVID, Neurological manifestation

## Abstract

Peripheral facial paralysis (PFP) has been shown to be a neurological manifestation of COVID-19. The current study presents two cases of PFP after COVID-19, along with a rapid review of known cases in the literature. Both case reports were conducted following CARE guidelines. We also performed a systematic review of PFP cases temporally related to COVID-19 using PubMed, Embase, and Cochrane Library databases on August 30, 2021, using a rapid review methodology. The two patients experienced PFP 102 and 110 days after COVID-19 symptom onset. SARS-CoV-2 RNA was detected in nasal samples through reverse-transcription real-time polymerase chain reaction (RT-qPCR) testing. Anosmia was the only other neurological manifestation. PFP was treated with steroids in both cases, with complete subsequent recovery. In the rapid review, we identified 764 articles and included 43 studies. From those, 128 patients with PFP were analyzed, of whom 42.1% (54/128) were male, 39.06% (50/128) female, and in 23 cases the gender was not reported. The age range was 18 to 59 (54.68%). The median time between COVID-19 and PFP was three days (ranging from the first symptom of COVID-19 to 40 days after the acute phase of infection). Late PFP associated with COVID-19 presents mild symptoms and improves with time, with no identified predictors. Late PFP should be added to the spectrum of neurological manifestations associated with the long-term effects of SARS-CoV-2 infection as a post COVID-19 condition.

## 1. Background

The COVID-19 pandemic has affected millions of people and threatened global public health [1, 2]. After months of the virus spreading, cases of post-COVID-19 syndrome began to emerge, characterized by signs and symptoms that develop during or after infection, continue for more than 12 weeks, and cannot be explained by any alternative diagnosis. Post-COVID-19 conditions are a wide range of new, returning, or ongoing health problems that people experience four or more weeks after being infected with the COVID-19 virus. Syndromes of post-viral manifestations have previously been reported in other pandemic events, such as severe acute respiratory syndrome (SARS) [3].

COVID-19 is a systemic disease that predominantly affects the respiratory system [4]. However, neurological changes may result from systemic complications of the disease, the direct effect of the virus on neurons, or an inflammation of the nervous system [5]. It is known that the SARS-CoV-2 infection is related to encephalopathies, central nervous system inflammatory syndromes, ischemic stroke, and peripheral neurological disorders; this relationship continues even after the acute phase of COVID-19 [6, 7]. Peripheral facial paralysis (PFP) occurs when there is a dysfunction of the facial nerve, usually affected in an inflammatory way, resulting in total or partial paralysis of facial movement. It is characterized by the involvement of the upper and lower areas of the face, which differentiates it from central peripheral paralysis, which only affects the lower portion [8]. It can be primarily referred to as Bell’s palsy, with an incidence rate of 15 to 30 cases per 100,000 inhabitants [9] or secondary, caused by metabolic, cerebrovascular, and mainly infectious factors [10].

This type of paralysis has been associated with viral infections, mainly herpes virus [8]. More recently, the association of PFP with SARS-CoV-2 has been reported [5, 11-13]. The diagnosis is made by observing the signs and symptoms. It is not necessary to employ imaging devices because, in this case, the benefits do not outweigh the harm when performing these tests [14].

In the present study, we report two cases of late PFP that occurred after COVID-19 in healthcare professionals, along with results of a review of the current literature evaluating PFP patients’ demographics, clinical characteristics, treatment, and outcomes of COVID-19.

## 2. Methods

The CARE guidelines [15] were used in the present case reports. Patients provided informed consent for publication of the cases, and the project was approved by the Institutional Review Board of Federal University de Mato Grosso do Sul, Brazil (protocol number 4.754.351).

### 2.1. Literature search strategy

A rapid systematic review was conducted, as recommended by Habby et al. [16]. The searches were conducted on PubMed, Embase, and Cochrane Library on August 30, 2021. The rapid review protocol was registered under number 242075 on PROSPERO (International Prospective Register of Systematic Reviews). The search strategy consisted of different combinations of the following search terms: (“COVID-19” OR “SARS-CoV-2”) AND (“Bell Palsy” [MeSH Terms] OR “Facial Paralysis”). The review did not have date or language restrictions. The articles in which PFP was characterized as Guillain-Barré syndrome (GBS) were excluded. The complete search strategies can be found in Additional file 1.

Additional file 1. Systematic review search strategy.

### 2.2. Data extraction

One reviewer conducted the screening by titles, abstracts, and descriptors to identify articles for analysis. A second reviewer screened all excluded abstracts, and any conflicts that arose were resolved. Data extraction was done by one reviewer and checked (“checked” value = “1”) for correctness and completeness by a second reviewer. A table was used to characterize the articles using the following information: article identification (by year, study site, and authors) and methodological profile of the article. When available, individuals’ data were included: patient demographics (age, gender, number of evaluated subjects, differential diagnosis, and probable diagnosis) and country of study origin. When the data were not available in the articles, they were requested from the author.

### 2.3. Statistical analyses

Descriptive analysis is expressed as frequencies and percentages for categorical variables and median and range for continuous variables. We present the results without meta-analysis due to the small numbers of studies currently available, considerable heterogeneity across studies and the high risk at bias that. Otherwise, the meta-analysis would produce a seemingly more accurate estimate than the underlying evidence is able to provide at this point in time.

### 2.4. Risk of bias assessment

Risk of bias assessment was carried out by one reviewer using the guidelines [15] recommended by the Joanna Briggs Institute. Full verification of all judgments was performed by a second reviewer.

## 3. Results

Our search yielded 127 cases of PFP after COVID-19 in 43 studies. Most of the cases were reported in Turkey (5.46%) and the USA (5.46%), followed by Spain (4.68%) and France (3.12%). Hospitals were the primary study settings in 22.65%. Males accounted for 42.19% of cases. For 23 cases, the gender of the patient was not reported. The youngest patient was 15 months old, and the oldest one was 90 years old. One patient was primigravida, at 39 weeks’ gestation (Table 1).

**Table 1.** Characteristics of included studies (n = 43)

First author’s name and year of publication	Country of origin	Study type	Study setting	Month and year of publication	Total number of subjects evaluated	Age	Sex (N)	Differential diagnosis	Probable diagnosis (all of them confirmed as positive for COVID-19)
Homma [11]	Japan	Case report	Hospital	May 2020	1	35 years	F	Rapid tests for influenza and streptococcus	COVID-19 pneumonia associated with facial nerve palsy and olfactory disturbance.
Ribeiro [12]	Brazil	Case report	Hospital	August 2020	1	26 years	M	Not reported	Facial paralysis.
Derollez [13]	France	Case report	Hospital	August 2020	1	57 years	F	Ganglioside antibodies, nuclear antibodies panel, rheumatoid factor, classical complement pathway, converting angiotensin enzyme, *Campylobacter jejuni* serology, HIV serology, HVA serology, HVB serology, HCV serology, CMV serology, VZV serology, EBV serology	Isolated cranial nerve deficit, especially facial nerve palsy, as a possible neurological manifestation due to COVID-19 infection.
Casas [17]	Spain	Case report	University hospital	June 2020	1	32 years	M	None	PFP secondary to neuritis of probable viral origin, related to SARS-CoV-2.
Ochoa-Fernández [18]	Spain	Case report	Emergency department	January 2021	1	6 years	F	None	PFP in pediatric age.
Doblan [19]	Turkey	Cross-sectional study	Hospital	March 2021	21	Range for 16 patients: 18-59 years Range for 5 patients: 60 years and over	M (11)/F (10)	Not reported	COVID-19, caused by the SARS-CoV-2 virus, commonly leads to cranial nerve symptoms.
Zain [20]	USA	Case report	Emergency room	April 2021	1	23 months old	F	Respiratory pathogen panel and cytomegalovirus RT-PCR; pathogen panel [serum Lyme titers; Epstein-Barr virus (EBV) IgM and IgG]	Peripheral nerve palsies associated with COVID-19 (brain MRI of the right cranial suggestive of neuritis).
Cabrera Muras [21]	Spain	Case report	University hospital	September 2020	1	20 years	M	EBV, enterovirus, herpes simplex virus (HSV), varicella-zoster, cytomegalovirus, parechovirus	SARS-CoV-2 infection preceded by upper respiratory symptoms, and evidence of coinfection with EBV.
Pinna [22]	USA	Case series	Large tertiary care academic center	June 2020	3	Not reported	Not reported	Not reported	Neurological manifestations in the setting of COVID-19.
Mehta [23]	Canada	Case report	Emergency department, University of Toronto	June 2020	1	36 years	M	None	Case of Bell’s palsy likely attributable to SARS-CoV-2.
Goh [24]	Singapore	Case report	Hospital (tertiary health care center)	August 2020	1	27 years	M	HSV, varicella-zoster virus (VZV), EBV, cytomegalovirus	Left lower motor neuron type facial nerve palsy associated with COVID-19.
Zammit [25]	UK	Retrospective review	Hospital (accident and emergency department)	September 2020	30	48 years ± 29 years	F (17)/M (13)	Not reported	Possibility of COVID-19 causing lower motor lower motor neuron VII^th^ cranial nerve palsy, with or without any preceding COVID-19 symptoms.
Chaumont [26]	France	Case series	Hospital	June 2020	4	60.5 ± 9.5 years	M	Not reported	Mixed central and peripheral disorders as a complication of severe COVID-19.
Bsales [27]	USA	Case report	Hospital (emergency department)	November 2020	1	2 years	M	Lyme disease, bacterial culture, meningitis encephalitis; HSV type-1 immunoglobulin antibody was reactive and HSV type-2 antibody was nonreactive; cytomegalovirus IgG antibody was positive and cytomegalovirus IgM was negative; blood EBV was undetected	Left-sided facial palsy in a previously healthy child who was simultaneously infected with SARS-CoV-2.
Mikaberidze [28]	Iran	Case report	Hospital	November 2020	1	60 years	M	Not reported	SARS-CoV-2-induced facial nerve palsy.
Bastola [29]	Nepal	Case report	Hospital	September 2020	1	48 years	M	Not reported	Bell’s palsy as a possible neurological complication of COVID-19 infection.
Decio [30]	Italy	Case report	Pediatric neurology department	December 2020	1	15 months	F	Serological tests for HSV 1, HSV 2, varicella zoster virus (VZV), EBV, cytomegalovirus, *Mycoplasma pneumoniae*, *Borrelia burgdorferi*	Facial palsy immune-mediated neurological complication of COVID-19, similar to transverse myelitis and GBS.
Roussel [31]	France	Case report	Hospital	July 2020	1	6 years	F	gram stain and culture, PCR for HSV, varicella, enterovirus, EBV, human herpes virus 6, herpes virus 8, adenovirus, mycoplasma, cryptococcus, toxoplasma nucleic acids	Children can display peripheral neuropathy, reminiscent of GBS or Miller-Fischer syndromes, with concomitant SARS-CoV-2 infection.
Kumar [32]	India	Case report	Hospital	February 2021	1	28 years	F	Guillain-Barré syndrome, Lyme disease, herpes virus, HIV serology	Neurocovid manifestation.
Neo [33]	Singapore	Case series	Hospital	February 2021	2	Patient 1: 25 years Patient 2: 35 years	M	Not reported	Possible association between isolated facial nerve palsy and SARS-CoV-2.
Dahl [34]	Norway	Case report	Hospital	December 2020	1	37 years	M	Cellulitis on the neck	The development of ganglioside antibodies during the course of the infection and the manifestation of Bell’s palsy only after he had recovered from the initial febrile infection support the conclusion that the palsy has autoimmune pathogenesis.
Islamoglu [35]	Turkey	Cross-sectional study	Hospital	February 2021	10	60 years or more	Not reported	Viral serology, especially HSV, HIV, VZV	COVID-19
Theophanous [36]	USA	Case report	Hospital	August 2020	1	6 years	M	HSV 1, HSV 2, VZV	COVID-19
Portela-Sánchez [37]	Spain	Cross-sectional study	Hospital	January 2021	1	Not reported	Not reported	Not reported	COVID-19
Lima [38]	Brazil	Case series	Hospital	September 2020	8	Average age: 36 years Range: 25-50 years	F (7)/M (1)	HSV	Peripheral facial palsy should be added to the spectrum of neurological manifestations associated with COVID-19.
Koh [39]	Singapore	Retrospective study	All the public healthcare institutions of the city	September 2020	5	Not reported	Not reported	Not reported	COVID-19.
Meppiel [40]	France	Retrospective study	Hospital	November 2020	1	Not reported	Not reported	Not performed	COVID-19.
Elibol [41]	Turkey	Retrospective study	Government hospital	August 2020	1	Not reported	Not reported	Not performed	COVID-19.
Taşlıderea [42]	Turkey	Case report	Emergency department	August 2020	1	51 years	F	Serology for HSV, cytomegalovirus	The patient had Melkersson-Rosenthal syndrome. COVID-19 infection, which was not previously included in the etiology of the disease, can now be considered among the causes of recurrence of the syndrome.
López-Blanco [43]	Spain	Case report	Hospital	May 2020	1	67 years	M	VZV	Varicella-zoster virus.
Kerstens [44]	Belgium	Case report	Emergency department	April 2021	1	27 years	M	EBV, HSV, VZV	Bilateral facial palsy associated with COVID-19.
Taouihar [45]	Morocco	Case report	University hospital center	August, 2021	2	Patient 1: 39 years Patient 2: 57 years	M (2)	Serologies of HSV, HZV, HIV, *Treponema pallidum* hemagglutination assay (TPHA) were performed and came back negative	SARS-CoV-2 viral infection.
Ozer [46]	Turkey	Case report	Clinic	June 2021	1	62 years	F	Not reported	PFP.
Egilmez [47]	Turkey	Retrospective observational study	Emergency department	May 2021	8	Average age: 50 years Range: 4-90 years	M (2)/F (6)	Not reported	Peripheral facial palsy can be encountered during the clinical course of COVID-19.
Hookham [48]	UK	Case report	Emergency department	May 2021	1	17 years	M	Not reported	Pediatric inflammatory multisystem syndrome.
Mutlu [49]	Turkey	Retrospective study	Hospital	April 2021	2	Not reported	Not reported	Not reported	Idiopathic PFP.
González-Castro [50]	Spain	Case report	Intensive Care Unit		2	Patient 1: 40 years Patient 2: 65 years	M and F	Not reported	PFP.
Tawfik [51]	Egypt	Cohort study	Hospital	March 2021	1	29 years	M	Not reported	Facial palsy.
Kaplan [52]	USA	Case report	Not reported	April 2021	1	48 years	F	Lyme disease	Bell’s palsy.
Hasibi [53]	Iran	Case report	Hospital	June 2021	1	52 years	M	Not reported	Facial palsy.
Aragão [54]	Brazil	Case control	Hospital and clinic	May 2021	1	40 years	M	Not reported	Facial palsy.
Figueiredo [55]	Portugal	Case report	Obstetric emergency department	July 2020	1	35 years	F (primigravida, 39 weeks’ gestation)	VZV and herpes virus infections unlikely; Lyme disease, HIV, vasculitis, sarcoidosis or other autoimmune diseases; neoplasm and cerebrovascular diseases.	Possible association between SARS-CoV-2 infection and Bell’s palsy.

Physical therapy based on facial exercises, ocular hydration and eye protection were indicated as non-drug treatment [17, 18, 32, 33, 55].

Three papers described the occurrence of Bell’s palsy in patients more than 30 days from their COVID-19 diagnosis [19, 39, 44]. Two articles were not included in the median calculation as they did not report this result. In the aforementioned two articles, the mean time between COVID-19 and PFP ranged from 7 to 12 days for three patients [47] and from 2 to 10 days in five patients [38]. In a study reporting 21 cases of nerve facialis [19], some patients presented associated diseases such as hypertension, diabetes mellitus, cardiac diseases, asthma, and Behçet’s disease, but the study did not report the distribution of these diagnoses. One patient was empirically treated with doxycycline for suspected borreliosis [34]. The other treatments as well as clinical features of patients with PFP after COVID-19 are presented in Table 2. In 38 (29.68%) cases of PFP, the information regarding the recovery was not described and, in 35 (27.35%) cases patients did not recover.

**Table 2.** Clinical features of patients with peripheral facial paralysis after COVID-19 (n = 127)

**Characteristics**	**N (%)**
**Male: Female** (54 : 50)	42.1 (39.7)
**Age**	
0 to 12 years	8 (6.2)
13 to 17 years	2 (1.5)
18 to 59 years	70 (54.6)
60 years or more	34 (26.5)
Not reported	13 (10.1)
**COVID-19 diagnostic method**	
Real-time RT-qPCR only	95 (74.2)
Serological test only	21(16.4)
Real-time RT-qPCR and serological test	8 (6.2)
Other (RT-PCR or of bronchoalveolar lavage fluid, real-time RT-qPCR or serological test or clinical history and the chest computed tomographic scan)	1 (0.7)
Not reported	2 (1.5)
**Time in days between COVID-19 and peripheral facial paralysis diagnosis (median)**	3 (0.4)
Associated diseases*	
Diabetes mellitus	9 (7.3)
Obesity	4 (3.1)
Hypertension	5 (3.9)
Obstructive sleep apnea syndrome	3 (2.3)
Varicella-zoster virus	2 (1.5)
Not reported	68 (53.1)
**Signs and symptoms**	
Headache	14 (10.9)
Anosmia	6 (4.6)
Ageusia	9 (7.3)
**House-Brackmann scale**	47 (36.7)
**Drug treatment for PFP**	
Corticosteroids	14 (10.9)
Corticosteroid and antiviral association	8 (6.2)
Intravenous immunoglobulin	4 (3.1)
Antiviral	
Lopinavir/ritonavir	1 (0.7)
Valaciclovir	7 (5.4)
Aciclovir	9 (7.0)
Without treatment	1 (0.7)
No pharmacological treatment reported	85 (66.4)
**Complete recovery of facial motility**	55 (42.9)

*Some patients presented with more than one associated disease.

## 4. Case Report 1

A female patient (37 years old; a resident of Campo Grande, MS, Brazil; married; nursing technician; brown) started experiencing a headache and runny nose on September 16, 2020 and developed diarrhea, myalgia, anosmia, and adynamia. The patient underwent oro-nasopharyngeal swab sample collection on September 18 for an RT-qPCR test using the Allplex 2019-nCoV real-time RT-qPCR kit (Seegene, Seoul, Korea). This multiplex assay uses oligonucleotides for the RdRP-gene (RNA-dependent RNA polymerase), N-gene (nucleocapsid), and E-gene (envelope) as viral targets, and RNase P-gene (ribonuclease P) as the internal control. CT values ≤ 38 were considered positive. CT values were 26 for E-gene, 28 for RdRP-gene, 36 for N-gene, and 26 for RNase P-gene, confirming the positive result for SARS-CoV-2 infection. A serology for the detection of anti-SARS-CoV-2 IgG antibodies by CLIA (Abbott) was performed on September 30, 2020 with a positive result.

 There was a progressive regression of symptoms and, on September 25, 2020, she became asymptomatic. One hundred and ten days after the onset of symptoms of COVID-19, the patient presented severe squeezing pain in the left ear for six hours, with an intensity of 10/10, which radiated to the temporal and zygomatic regions. The pain ceased with the use of dipyrone (the patient could not recall the dosage). The next morning, she noticed lip rhyme deviation, went to an emergency care unit close to her home, and received a clinical diagnosis of Bell’s palsy on the left side. She had no history of previous facial paralysis, labial or genital herpes, or other comorbidities. According to the House-Brackmann classification system, the damage resulted in moderately severe dysfunction in the patient (Grade IV).

The following drugs were prescribed orally: acyclovir 400 mg every four hours for 10 days; prednisone 60 mg for the first five days; prednisone 30 mg for the final five days; and prednisone 15 mg for five days. The patient underwent facial physiotherapy, with progressive improvement in her condition. Symptoms ceased in 15 days. The following laboratory tests were performed: serology for HIV (non-reactive), brucellosis 1^st^ and 2^nd^ samples (non- reactive), cytomegalovirus IgG (reactive) and IgM (non- reactive), Lyme IgG (non- reactive), and Lyme IgM (non- reactive). No imaging exams were performed.

## 5. Case Report 2

A female patient (39 years old; resident of Campo Grande, MS, Brazil, for 28 years; married; nurse; white) received a confirmed diagnosis of SARS-CoV-2 infection on September 19, 2020 through a real-time RT-qPCR test, performed for infection surveillance. The Allplex 2019-nCoV real-time RT-qPCR kit (Seegene, Seoul, Korea) was used and the CT values were 29 for E-gene, 29 for RdRP-gene, 30 for N-gene, and 28 for RNase P-gene. The patient was asymptomatic for four days after the test was collected, when she declared that she was experiencing myalgia, nausea, adynamia, nasal congestion, anosmia, headache, and cough. On September 27, 2020, she was clinically evaluated and complained of right chest pain, with a diagnosis of pneumonia; prednisolone 10 mg was prescribed for five days. On October 9, 2020, the patient manifested tiredness, fatigue, headache, and presented tachycardia on physical examination. A beta-blocker (bisoprolol 2.5 mg daily) was prescribed due to tachycardia, with partial improvement. Subsequently, the patient continued to report anosmia and migraine. After 103 days since the COVID-19 diagnosis, the patient had mild paralysis on the right side of her face, and she was clinically diagnosed with Bell’s palsy. According to the House-Brackmann classification system, the damage resulted in mild dysfunction (Grade 2). The patient had no history of previous facial paralysis, labial or genital herpes, or comorbidities. 

The patient took 300 mg of dipyrone monohydrate, 35 mg of orphenadrine citrate, and 50 mg of anhydrous caffeine, orally, every eight hours for five days for pain and relief of muscle contracture of the mandible contralateral to the paralysis. She also underwent facial physiotherapy at home. In consultation with an otolaryngologist, due to tinnitus and right ear pain on December 8, 2020, she started using inhaled corticosteroids (fluticasone furoate nasal spray, 27.5 mcg) for 14 days, and noticed an improvement in her migraine and otological symptoms. Her facial paralysis lasted for 10 days, and her symptoms progressively improved with partial recovery up to the time of this report.

A cranial MRI performed to investigate persistent migraine did not show any changes. The following serology tests were performed: HIV (non-reactive), brucellosis 1^st^ and 2^nd^ samples (non-reactive), cytomegalovirus IgG (reactive), IgM (non-reactive), Lyme IgG (non-reactive), and Lyme IgM (non-reactive).

## 6. Discussion

This study describes two cases of late PFP after confirmed COVID-19 infections. In the literature search, we retrieved 43 articles reporting patients with acute paralysis or idiopathic facial weakness associated with SARS-CoV-2 infection. Numerous late cases of Bell’s palsy weeks after the COVID-19 diagnosis have likely not been published as related to Sars-CoV-2. In this case, it is necessary to take into account the publication bias [56]. Although a problem may be relevant to the scientific community, many studies are published or not depending on the results.

Although facial paralysis has no gender predilection, our two reports involved female patients, as indicated in recent reports on the subject [11, 12, 24, 55]. The pathophysiology of the disease may be related to increased viral replication and dissemination in the axon, leading to demyelination and inflammation [57]. The severity of this condition can be measured by the addition of more neurological events, intensification of initial symptoms, ocular symptoms, and incomplete recovery after three months [5].

In general, the cases we have reported had benign courses with some degree of facial involvement. Patient 1 was classified as grade III, according to an assessment of facial movement using the House-Brackmann scale [58], with no movement in the forehead, incomplete eye closure with effort, and asymmetry of the mouth with maximum effort. Patient 2 was classified as grade II, with moderate to good forehead function, complete eye closure with minimal effort, and mild asymmetry in the mouth. As a result of this systematic review, most cases were grade III [17, 25, 38, 44], which means that a large number of patients affected by paralysis had moderate symptoms.

The cases presented here diverge from the literature regarding temporality and the symptom itself; in other reports, paralysis has generally been described as the only symptom or as an initial symptom [11, 18, 20, 22, 33, 36, 38, 39, 55].

The cases described in this work presented other manifestations, such as cough, body pain, and myalgia. The paralysis occurred four months after symptoms and the COVID-19 diagnosis. The persistence of symptoms after the acute phase of COVID-19 has been discussed and the term *long COVID* is used for this purpose: when there are lasting effects of the infection after three weeks from the time of diagnosis. The virus can cause permanent damage to various organs, such as the heart, lung, and brain [59]. Therefore, we classify the cases presented here as long COVID.

Except for the anosmia presented by the two patients, the association of other neurological manifestations concomitant with the paralysis was not observed. In recent reports, the involvement of the facial nerve - during or after COVID-19 infection - was associated with the absence of deep tendon reflexes, ataxia, hypoesthesia, or paresthesia in the upper and lower limbs, being characteristic signs of Guillain-Barré syndrome (GBS) [60-63].

The World Health Organization, in April 2020, determined that cases with acute disseminated encephalomyelitis, GBS, and other acute neuropathies could be associated with SARS-CoV-2 infection [5]. Our reported cases do not coincide with the concepts of probable and possible association described in the literature [64]. However, this definition of temporal association can be changed as new events have been reported throughout the pandemic [65].

Additionally, no changes in taste have been reported, although ageusia has emerged as one of the common symptoms of COVID-19 and these manifestations are present in cases of peripheral paralysis, when the topography of the lesion reaches the chorda tympani nerve, a branch of the facial nerve [66].

Treatment for paralysis aims to improve the facial mimicry of these patients and prevent sequelae. There is evidence that physiotherapy sessions can offer benefits for recovering facial mobility and improving the functionality of facial expressions, accelerating the recovery process [67]. In addition to physiotherapy sessions, the study patients used corticosteroids. One corticosteroid was associated with an antiviral. In the review, eight patients received the association of antivirals and corticotherapy, in consideration of a possible HSV infection [24, 32, 33, 36, 38]. Another 17 patients used antiviral drugs such as lopinavir/ritonavir, valacyclovir, and acyclovir to treat paralysis [11, 25, 51, 53].

Finally, concerning the duration of symptoms mentioned in other articles on the subject, which ranged from six days for complete recovery [11] to 30 days for partial recovery [38], the total recovery of the facial mimicry of patients described in our report has an uncertain but promising prognosis.

There is a notable increase in described neurological manifestations including facial paralysis cases, with an onset time that can vary from two days to 60 days. Different from the observed in the rapid review, the two cases are considered late peripheral facial paralysis after COVID-19 because the onset of symptoms occurs more than 100 days after the onset of the disease.

The limitations of our study are inherent to the types of research included in the rapid review, which do not describe their limitations, making it difficult to compare with cases related globally. Additionally, the difficulty in differentiating isolated paralysis and facial paralysis associated with GBS is detected in the included articles. In our report, it was not possible to perform HSV serology due to lack of a kit in the reference laboratory. Also, MRIs were not performed in the acute period of the onset of facial paralysis. Due to the low CT, it was not possible to perform genetic sequencing of the virus.

## 7. Conclusion

In conclusion, cases of peripheral facial paralysis have been described in the literature, but they are limited to acute cases with descriptions of up to 60 days in duration. There is a wide variety of clinical protocols and treatments. We report two cases of PFP after confirmed COVID-19 infection, both presenting some symptoms related to COVID-19, with late neurological alteration. Considering that these two cases had no history of labial or genital herpes and that both cases had positive serology (IgG) for cytomegalovirus, this late symptomatology is a hypothesis that it is caused by Sars-Cov-2. The benign course of the disease demonstrated the diversity of complications caused by SARS-CoV-2 and the need for outpatient follow-up to observe possible late manifestations of FPF.

## Supplementary material

The following online material is available for this article:

Additional file 1 Systematic review search strategy

.
