# Supplementary material for: Late peripheral facial paralysis after COVID-19: a rapid systematic review and two case reports
Source: J Venom Anim Toxins Incl Trop Dis. 2022 Oct 17;28:e20220020. doi: 10.1590/1678-9199-JVATITD-2022-0020 (PMC9581522; doi:10.1590/1678-9199-JVATITD-2022-0020)
Supplement: Additional file 1 [file 1678-9199-jvatitd-28-e20220020-s1.pdf]

J Venom Anim Toxins incl Trop Dis

https://doi.org/10.1590/1678-9199-JVATITD-2022-0020

Supplementary material to: "Late peripheral facial paralysis after COVID-19: a rapid systematic review and two case reports"

Additional file 1. Systematic review search strategy

Number	Combiners	Terms
**1**	**COVID**	("COVID-19" OR "2019 novel coronavirus disease" OR "COVID19" OR "COVID-19 pandemic" OR "SARS-CoV-2 infection" OR "COVID-19 virus disease" OR "2019 novel coronavirus infection" OR "2019-nCoV infection" OR "Coronavirus disease 2019" OR "Coronavirus disease-19" OR "2019-nCoV disease" OR "COVID-19 virus infection" OR "Coronavirus infection" OR "Severe acute respiratory syndrome coronavirus 2" OR "2019-nCoV" OR "Wuhan coronavirus" OR "SARS-CoV-2" OR "2019 novel coronavirus" OR "COVID-19 virus" OR "Coronavirus disease 2019 virus" OR "COVID19 virus" OR "Wuhan seafood marked pneumonia virus")
**2**	**Peripheral Facial Paralysis**	("Bell Palsy" OR "Bell Palsies" OR "Idiopathic Facial Paralyses" OR "Idiopathic Facial Paralysis" OR "Acute Inflammatory Facial Neuropathy" OR "Idiopathic Acute Facial Neuropathy" OR "Bell's Palsy" OR "Bell's Palsies" OR "Bell's Palsy" OR "Acute Idiopathic Facial Neuropathy" OR "Herpetic Facial Paralysis" OR "Herpetic Facial Paralyses" OR "Facial Palsy" OR "Facial Palsy, Lower Motor Neuron" OR "Facial Palsy, Upper Motor Neuron" OR "Facial Paralysis, Peripheral" OR "Facial Paresis" OR "Hemifacial Paralysis" OR "Lower Motor Neuron Facial Palsy" OR "Upper Motor Neuron Facial Palsy" OR "Facial Paralysis")

	Terms
**Pubmed**	(“COVID-19” OR “2019 novel coronavirus disease” OR “COVID19” OR “COVID-19 pandemic” OR “SARS-CoV-2 infection” OR “COVID-19 virus disease” OR “2019 novel coronavirus infection” OR “2019-nCoV infection” OR “Coronavirus disease 2019” OR “Coronavirus disease-19” OR “2019-nCoV disease” OR “COVID-19 virus infection” OR “Coronavirus infection” OR “Severe acute respiratory syndrome coronavirus 2” OR “2019-nCoV” OR “Wuhan coronavirus” OR “SARS-CoV-2” OR “2019 novel coronavirus” OR “COVID-19 virus” OR “Coronavirus disease 2019 virus” OR “COVID19 virus” OR “Wuhan seafood marked pneumonia virus”) AND (“Bell Palsy” OR “Bell Palsies” OR “Idiopathic Facial Paralyses” OR “Idiopathic Facial Paralysis” OR “Acute Inflammatory Facial Neuropathy” OR “Idiopathic Acute Facial Neuropathy” OR “Bell's Palsy” OR “Bell's Palsies” OR “Bells Palsy” OR “Acute Idiopathic Facial Neuropathy” OR “Herpetic Facial Paralysis” OR “Herpetic Facial Paralyses” OR “Facial Palsy” OR “Facial Palsy, Lower Motor Neuron” OR “Facial Palsy, Upper Motor Neuron” OR “Facial Paralysis, Peripheral” OR “Facial Paresis” OR “Hemifacial Paralysis” OR “Lower Motor Neuron Facial Palsy” OR “Upper Motor Neuron Facial Palsy” OR “Facial Paralysis”)
**Embase**	('covid-19'/exp OR 'covid-19' OR '2019 novel coronavirus disease'/exp OR '2019 novel coronavirus disease' OR 'covid19'/exp OR 'covid19' OR 'covid-19 pandemic' OR 'sars-cov-2 infection'/exp OR 'sars-cov-2 infection' OR 'covid-19 virus disease' OR '2019 novel coronavirus infection'/exp OR '2019 novel coronavirus infection' OR '2019-ncov infection'/exp OR '2019-ncov infection' OR 'coronavirus disease 2019'/exp OR 'coronavirus disease 2019' OR 'coronavirus disease-19'/exp OR 'coronavirus disease-19' OR '2019-ncov disease'/exp OR '2019-ncov disease' OR 'covid-19 virus infection' OR 'coronavirus infection'/exp OR 'coronavirus infection' OR 'severe acute respiratory syndrome coronavirus 2'/exp OR 'severe acute respiratory syndrome coronavirus 2' OR '2019-ncov'/exp OR '2019-ncov' OR 'wuhan coronavirus'/exp OR 'wuhan coronavirus' OR 'sars-cov-2'/exp OR 'sars-cov-2' OR '2019 novel coronavirus'/exp OR '2019 novel coronavirus' OR 'covid-19 virus'/exp OR 'covid-19 virus' OR 'coronavirus disease 2019 virus' OR 'covid19 virus' OR 'wuhan seafood marked pneumonia vírus') AND ('bell palsy'/exp OR 'bell palsy' OR 'bell palsies' OR 'idiopathic facial paralyses' OR 'idiopathic facial paralysis'/exp OR 'idiopathic facial paralysis' OR 'acute inflammatory facial neuropathy'/exp OR 'acute inflammatory facial neuropathy' OR 'idiopathic acute facial neuropathy'/exp OR 'idiopathic acute facial neuropathy' OR 'bell’s palsy' OR 'acute idiopathic facial neuropathy'/exp OR 'acute idiopathic facial neuropathy' OR 'herpetic facial paralysis'/exp OR 'herpetic facial paralysis' OR 'herpetic facial paralyses' OR 'facial palsy'/exp OR 'facial palsy' OR 'facial palsy, lower motor neuron' OR 'facial palsy, upper motor neuron' OR 'facial paralysis, peripheral' OR 'facial paresis'/exp OR 'facial paresis' OR 'hemifacial paralysis' OR 'lower motor neuron facial palsy' OR 'upper motor neuron facial palsy' OR 'facial paralysis'/exp OR 'facial paralysis')
**Cochrane**	‘COVID’ OR ‘Peripheral Facial Paralysis’ AND ‘Cochrane system word variation’
